# Tau underlies synaptic and cognitive deficits for type 1, but not type 2 diabetes mouse models

**DOI:** 10.1111/acel.12919

**Published:** 2019-02-27

**Authors:** Laura Trujillo‐Estrada, Cassidy Nguyen, Celia da Cunha, Lena Cai, Stefania Forner, Alessandra C. Martini, Rahasson R. Ager, Gilberto Aleph Prieto, Carl W. Cotman, David Baglietto‐Vargas, Frank M. LaFerla

**Affiliations:** ^1^ Institute for Memory Impairments and Neurological Disorders University of California Irvine California; ^2^ Department of Neurobiology and Behavior University of California Irvine California; ^3^ Department of Neurology University of California Irvine California

**Keywords:** Alzheimer’s disease, cognition, dendritic spines, diabetes mellitus, synaptic deficit, tau

## Abstract

Diabetes mellitus (DM) is one of the most devastating diseases that currently affects the aging population. Recent evidence indicates that DM is a risk factor for many brain disorders, due to its direct effects on cognition. New findings have shown that the microtubule‐associated protein tau is pathologically processed in DM; however, it remains unknown whether pathological tau modifications play a central role in the cognitive deficits associated with DM. To address this question, we used a gain‐of‐function and loss‐of‐function approach to modulate tau levels in type 1 diabetes (T1DM) and type 2 diabetes (T2DM) mouse models. Our study demonstrates that tau differentially contributes to cognitive and synaptic deficits induced by DM. On one hand, overexpressing wild‐type human tau further exacerbates cognitive and synaptic impairments induced by T1DM, as human tau mice treated under T1DM conditions show robust deficits in learning and memory processes. On the other hand, neither a reduction nor increase in tau levels affects cognition in T2DM mice. Together, these results shine new light onto the different molecular mechanisms that underlie the cognitive and synaptic impairments associated with T1DM and T2DM.

## 1 INTRODUCTION

Neurodegenerative disorders are a heterogeneous group of diseases that share an underlining progressive loss of neurons and synaptic connections, of which no cure has been found (Dickstein et al., 2007). Many of these diseases, including Alzheimer's disease (AD), induce neurodegenerative and pathological processes via the abnormal accumulation of the microtubule‐associated protein tau (MAPT). These disorders, generally called tauopathies, are characterized by the abnormal metabolism of tau proteins, which can lead to the formation of intracellular neurofibrillary tangles (NFTs) and the disruption on synaptic activity (Forner, Baglietto‐Vargas, Martini, Trujillo‐Estrada, & LaFerla, 2017). For example, relevant studies in AD have demonstrated that tau pathology correlates with the severity of dementia and neuronal loss in human cases (Riley, Snowdon, & Markesbery, 2002), and multiples findings in AD animal models have shown that synaptic and cognitive deficits depend on tau (Ittner et al., 2010). Collectively, these findings suggest that tau is a critical component in the manifestation of the neurodegenerative pathology associated with many brain disorders.

Interestingly, new studies have shown that diabetes mellitus (DM), the most common metabolic disorder (Sarah et al., 2004), increases the risk of developing several different neurodegenerative disorders, and predisposes individuals to cognitive decline (Yuan & Wang, 2017). Moreover, emerging epidemiological studies have found that DM has a profound impact on AD risk (Sutherland, Lim, Srikanth, & Bruce, 2017). *Postmortem* studies on the brains of diabetic patients have shown increased amyloid‐β and hyperphosphorylated tau deposition compared to age‐matched controls (Valente, Gella, Fernàndez‐Busquets, Unzeta, & Durany, 2010). In addition, diabetic AD patients exhibit more robust pathological changes compared to nondiabetic AD patients (Valente et al., 2010). Due to the expected increase in the number of people afflicted with DM in the upcoming decades, and the resulting pathological consequences on cognitive processes, it is critical to dissect the cellular and molecular mechanisms by which diabetes impacts cognition.

Interestingly, previous work from our laboratory has found that reducing tau levels mitigates memory and synaptic impairments in T1DM‐induced mice, suggesting that T1DM requires the presence of tau to trigger cognitive deficits (Abbondante et al., 2014). In the current study, we test the hypothesis that tau is required for the cognitive dysfunction associated with T2DM, and further investigate whether tau is a critical mediator in the synaptic/memory dysfunction associated with T1DM. Our results show that tau has a differential impact on the cognitive and synaptic deficits induced by T1DM and T2DM.

## 2 RESULTS

### 2.1 Diabetes increases tau pathology in human samples

**Figure 1:**
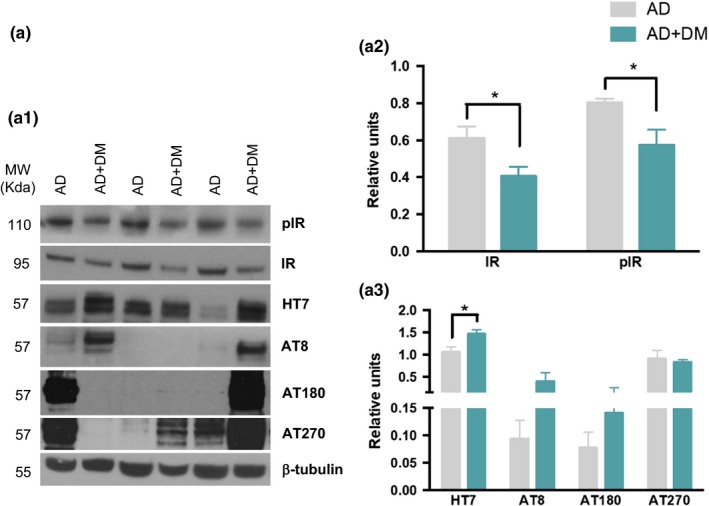
Diabetic condition increases tau pathology in AD human samples. (a) Immunoblot analyses of insulin receptor (IR), phosphor‐insulin receptor (pIR), total tau (HT7), pSer199/202 tau (AT8), pThr231 (AT180), and pThr181 (AT270) of protein extracts from superior frontal gyrus of human AD patients with and without diabetes, are shown in alternating lanes (a1). (a2) Quantification normalized to β‐tubulin for IR and pIR. Diabetic conditions in AD patients impair insulin receptor, reducing the levels of both insulin receptor and its phosphorylated form (unpaired *t* test, *p < 0.05, *n* = 5 per group). (a3) The quantification of HT7, AT8, AT180, and AT270 normalized to β‐tubulin and expressed as relative units, revealed an increase in tau pathology in AD patients with diabetes (unpaired *t* test, *p < 0.05, *n* = 5 per group). The values represent means ± *SEM*

To investigate the effects of diabetes on tau pathology in AD, we analyzed tau and phosphorylated tau levels in human synaptosomes from AD patients with and without diabetes (Figure 1). First, we sought to verify the patient's diabetic clinical diagnosis by testing for changes in the insulin receptor (IR) (Frölich, Blum‐Degen, Riederer, & Hoyer, 1999). Western blot (WB) analysis showed further impairments in IR and phosphor‐insulin receptor (pIR) levels in diabetic AD samples compared to AD samples without diabetes (Figure 1a1, a2). Next, we investigated the effect of diabetes on tau pathology. WB analysis revealed a significant increase in total tau levels (HT7) in diabetic AD samples (Figure 1a1, a3) and an increase in tau phosphorylation at residues Ser202/Thr205 and pThr231 (recognized by the AT8 and AT180 antibody respectively), though the increase in tau phosphorylation was not significant. These data suggest that diabetes accelerates tau pathology in AD patients.

### 2.2 Metabolic features of type 1 and type 2 diabetic mouse models

To determine whether a diabetic condition was induced after streptozotocin (STZ) treatment, we measured for changes in body weight, blood glucose, glycated hemoglobin (A1c), blood insulin, insulin receptor expression, and phosphor‐IR expression in nontransgenic (Ntg) and wild‐type human tau (htau) mice (Figure S1). This analysis showed that STZ reduces body weight (Figure S1A), while it increases glucose and A1c levels (Figure S1B–C), thus confirming T1DM induction. Insulin concentration and pIR levels were decreased in diabetic mice; however, no changes were observed in the expression of the IR (Figure S1D–E). Therefore, STZ successfully induced a diabetic condition similarly in Ntg and htau mice.

For type 2 diabetes mice, we also measured for changes in the body weight, glucose level, A1c, insulin, and IR for db/db‐htau mice (Figure S2A–E), and A1c and insulin concentration for db/db‐tau knock‐out (db/db‐tauKO) mice (Figure S2F–G), showing a high level of glucose, A1c, and insulin levels in db/db, db/db‐htau, and db/db‐tauKO mice. Recent findings showed changes in glucose and insulin levels in the tauKO mice (Wijesekara, Gonçalves, Ahrens, De Felice, & Fraser, 2018). However, we do not observe any difference in these levels between Ntg and tauKO mice. These differences could be related to the age of the mice at which we performed the study (8 weeks vs. 5 months old).

### 2.3 T1DM causes hippocampal cognitive deficits through tau‐dependent mechanisms

**Figure 2:**
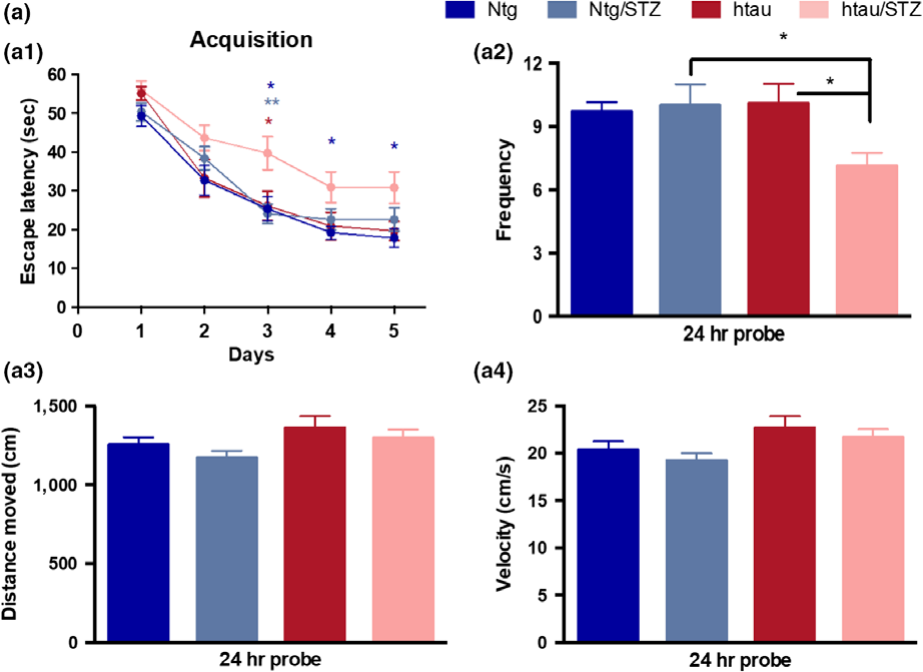
Streptozotocin treatment induces hippocampal cognitive impairment through tau‐dependent mechanisms. (a) Mice were trained on the spatial reference version of the MWM at 15 months of age. Acquisition curves (a1) are shown for the 5 days of training on the MWM. Two‐way ANOVA: trials [*F*(4, 313) = 62.62, *p* < 0.0001], treatment [*F*(3, 313) = 12.54, *p* < 0.0001], and interaction [*F*(12, 313) = 0.6436, *p* = 0.8043], Tukey's multiple comparisons test, **p < 0.01, *p < 0.05 (significance indicated vs. htau/STZ for the different groups, blue for Ntg, light blue for Ntg/STZ, and red for htau). (a2) Frequency of Ntg, Ntg/STZ, htau, and htau/STZ groups. Time to reach the platform is reduced in htau/STZ. One‐way ANOVA, *p = 0.0190, *F*(3, 50) = 3.632, Tukey's multiple comparisons test, *p < 0.05. Distance moved (a3) and velocity (a4) showed no differences between groups. *n* = 12–16 per group. The values represent means ± *SEM*

To assess whether tau facilitates T1DM‐induced cognitive decline, we evaluated spatial memory in vehicle and STZ‐diabetic Ntg and htau mice by using the Morris water maze (MWM) test (Figure 2a). htau/STZ‐treated mice showed significant impairments in learning during acquisition compared to Ntg, Ntg/STZ, and htau mice (Figure 2a1). No differences in learning were detected between Ntg/STZ and Ntg or htau mice. Indeed, Ntg, Ntg/STZ, and htau mice reached criterion (under 25 s to reach the submerge platform) in 4 days, whereas htau/STZ mice did not reach the platform (Figure 2a1). Therefore, induction of a T1DM‐like condition impaired spatial learning only in htau mice, which suggest that htau overexpression is required for T1DM to induce learning deficits.

In addition, mice were tested 24 hr after the last training trial to evaluate memory. The results show that htau/STZ mice possess memory impairments, as determined by a significant decrease in the time to the target area (Figure 2a2). Importantly, the cognitive impairment observed in htau/STZ mice was not attributed to motor deficits, since no changes were noted for traveled distance and velocity (Figure 2a3, a4). These data suggest that T1DM induced severe hippocampal learning and memory deficits through tau‐dependent mechanisms.

### 2.4 T1DM affects dendritic spines and impairs synaptic function via tau‐dependent mechanisms

**Figure 3:**
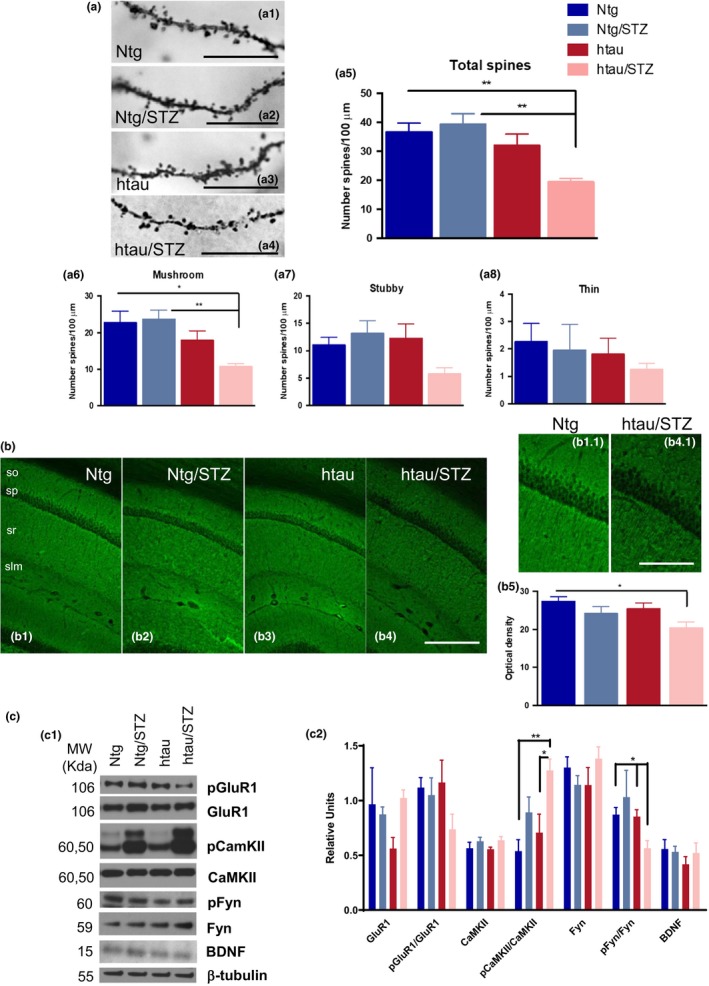
htau is necessary for STZ to alter dendritic spines number and impairs memory‐related intracellular signaling and levels of synaptic‐related proteins. (a) Dendritic spines analysis in Ntg, Ntg/STZ, htau, and htau/STZ. Light microscopic images of radiatum layer in CA1 subfield in Ntg (a1), Ntg/STZ (a2), htau (a3), and htau/STZ (a4). (a5) Stereological quantification showed significant decrease for htau/STZ group in total (47.13%  3.16 vs. Ntg, and 50.67%  2.95 vs. Ntg/STZ. One‐way ANOVA, **p = 0.0015, *F*(3, 16) = 8.294, Tukey's multiple comparisons test, **p < 0.01), and mushroom (52.81%  3.65 vs. Ntg, and 54.64%  3.50 vs. Ntg/STZ. One‐way ANOVA, **p = 0.0059, *F*(3, 16) = 6.063, Tukey's multiple comparisons test, **p < 0.01, *p < 0.05) spines. *n* = 4–6 per group. (b1‐b4) Synaptophysin confocal microscopic images of hippocampal CA1 layer in Ntg (b1), Ntg/STZ (b2), htau (b3), and htau/STZ (b4). Details of b1 and b4 in b1.1 and b4.1, respectively. Quantitative analysis of these images revealed a reduction in the optical density of synaptophysin in the htau/STZ group compared to Ntg (25.43%  5.61. One‐way ANOVA, *p* = 0.0572, *F*(3, 16) = 3.083, Tukey's multiple comparisons test, *p < 0.05. *n* = 4–6 per group. (c) Immunoblot analysis of GluR1, pGluR1, CamKII, pCaMKII, Fyn, pFyn, and BDNF of protein extracts from hippocampal synaptosome fractions of Ntg, Ntg/STZ, htau, and htau/STZ is shown in alternate lanes (c1). (c2) Quantification normalized to β‐tubulin for GluR1, CaMKII, Fyn, and BDNF, normalized to GluR1 for pGluR1, to CaMKII for pCaMKII and to Fyn for pFyn and expressed as relative units, showed a significant reduction in pFyn/Fyn (35.20%  7.91 vs. Ntg, and 33.82%  8.08 vs. htau) and a significant increase in pCaMKII/CaMKII (45.05%  4.54 vs. Ntg, and 59.16%  5.97 vs. htau) for the htau/STZ group (pFyn/Fyn *t* test Mann–Whitney, *p < 0.05. pCamKII/CamKII one‐way ANOVA, **p = 0.0044, *F*(3, 30) = 5.378, Tukey's multiple comparisons test, **p < 0.01, *p < 0.05. *n* = 4–6 per group). The values represent means ± *SEM*. Scale bars: 10 µm (a2–a5), 250 µm (b1–b4), 125 µm (b1.1–b4.1). so: stratum oriens, sp: stratum pyramidale, sr: stratum radiatum, slm: stratum lacunosum‐moleculare

Dendritic spines are specialized structures whose plasticity underlie learning and memory processes (Rochefort & Konnerth, 2012). Thus, it is plausible that the impairments in learning and memory observed in htau/STZ mice are associated with structural and morphological alterations of spines. To investigate this, we performed Golgi staining and stereological quantification of dendritic spines, as well as we measured the number of presynaptic puncta by using the antibody synaptophysin in the stratum radiatum (sr) of CA1 hippocampal area. Stereological quantification indicated that htau/STZ mice have significant deficits in dendritic spine density (Figure 3a1–a5), particularly in mushroom‐type spines (Figure 3a6). Consistent with this result, we detected a significant reduction in synaptophysin optical density in htau/STZ mice compared to Ntg (Figure 3b). We also observed a trend reduction in synaptophysin and profilin levels by WB (Figure S3). These data support the idea that T1DM further accelerate synaptic alterations in htau mice.

We next analyzed the levels of the synaptic‐related proteins GluR1, calcium/calmodulin‐dependent protein kinase II (CaMKII), Fyn, and brain‐derived neurotrophic factor (BDNF) by WB in hippocampal synaptosomes. Changes in the levels and activity of these synaptic markers have been associated with memory impairments in AD (Chen et al., 2014; Ghosh & Giese, 2015; Trepanier, Jackson, & MacDonald, 2012). WB analysis revealed a significant decrease in the steady‐state levels of the phosphorylated form of Fyn, and an increase in the phosphorylated form of CaMKII, in htau/STZ mice (Figure 3c). The phosphorylated form of GluR1 was also found to be decreased in htau/STZ mice, although the difference did not reach significance (Figure 3c). We also evaluated other factors by which tau could induce these deficits in htau/STZ mice. With this regard, we investigated the inflammatory response, which it has been described that play an important role in synaptic and cognitive processes (Donzis & Tronson, 2014), although no difference in any measured cytokines was observed in T1DM mice (Figure S4). Thus, these results indicate that tau did not affect the synapses through alterations in the inflammatory response in our T1DM model. Overall, our data suggest that changes in Fyn and CaMKII may be a major downstream causative factors for tau to induce synaptic and cognitive impairments in T1DM model.

### 2.5 **Tau phosphorylation in T1DM is associated with GSK3**β

**Figure 4:**
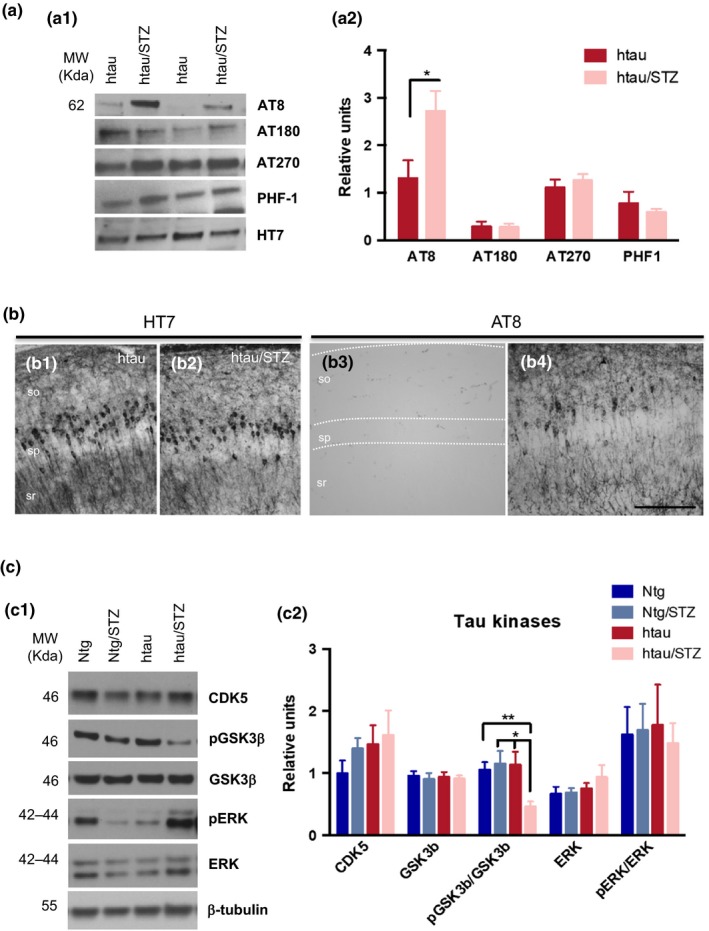
Streptozotocin increases AT8 tau phosphorylation in htau mice. (a) Immunoblot analyses of phosphor‐tau epitopes, including pSer199/202 tau (AT8), pThr231 (AT180), pThr181 (AT270), and pSer396/404 (PHF‐1) of protein extracts from hippocampal synaptosome fraction of htau and htau/STZ mice, are shown in alternating lanes (a1). (a2) Quantification normalized to HT7 and expressed as relative units. A significant increase was observed in p‐tau epitopes at Ser199/202 (54.80%  7.49, unpaired *t* test, *p < 0.05. *n* = 6–8 per group). (b) Light microscopic images stained with HT7 (b1–b2) and AT8 (b3–b4) in htau (b1 and b3) and htau/STZ (b2 and b4). No differences were detected in HT7 staining whereas in AT8 revealed an increase in phosphorylated tau in htau/STZ compared to htau mice. (c) Immunoblot analyses of CDK5, ERK, pERK, GSK3β, and pGSK3β of protein extracts from hippocampal synaptosomes of Ntg, Ntg/STZ, htau, and htau/STZ mice are shown in alternating lanes (c1). (c2) Quantification normalized to β‐tubulin and expressed as relative units, for CDK5, ERK, and GSK3β, normalized to ERK for pERK, and normalized to GSK3β for pGSK3β. Streptozotocin treatment does not alter CDK5, ERK, pERK, and GSK3β kinases. However, there is a reduction in pGSK3β in htau/STZ (56.20%  7.96 vs. Ntg, 60.01%  7.27 vs. Ntg/STZ, and 59.35%  7.39 vs. htau) compared to Ntg, Ntg/STZ, and htau mice (one‐way ANOVA, *p = 0.0114, *F*(3, 16) = 5.111, Bonferroni's multiple comparisons test, **p < 0.01, *p < 0.05. *n* = 5–6 per group). The values represent means ± *SEM*. Scale bars: 100 µm (b1–b4), so: stratum oriens, sp: stratum pyramidale, sr: stratum radiatum

Next, we investigated the effect of T1DM on tau phosphorylation in the htau mice. Tau is a cytoskeleton protein that contributes to microtubule stability. When hyperphosphorylated, tau lacks the affinity for the microtubules and promotes their destabilization (Wang & Mandelkow, 2015). Hyperphosphorylated tau is located in both the postsynaptic and presynaptic compartment where it might be involved in synaptic dysfunction (Forner et al., 2017). We reasoned that T1DM may lead to cognitive deficits by contributing to the htau hyperphosphorylated state. Therefore, we investigated the effect of STZ treatment on tau phosphorylation. WB analysis in hippocampal synaptosomes revealed a significant increase in tau phosphorylation at residues Ser202/Thr205 (AT8) in the htau/STZ‐treated compared to htau mice (Figure 4a). In contrast, neither steady‐state tau (HT7) nor phosphor‐tau species recognized by AT180 (Thr231), AT270 (Thr181), or PHF1 (Ser396/404) were altered by T1DM (Figure 4a). Immunohistochemistry analysis showed no difference in HT7 staining between htau and htau/STZ (Figure 4b1–b2); however, a different staining pattern was observed for AT8 (Figure 4b) with an increase in AT8 immunostaining in htau/STZ mice (Figure 4b4) compared to htau mice (Figure 4b3). These results indicate that STZ treatment led to tau hyperphosphorylation at Ser202/Thr205 epitope which can contributes to the cognitive impairment observed in T1DM‐like htau mice.

It is well known that the phosphorylation of tau is related to the activities of its kinases and phosphatases (Forner et al., 2017). Therefore, we next examined the levels of total and activated forms of specific kinases. Among all the tau kinases, glycogen synthase kinase‐3‐beta (GSK3β), cyclin‐dependent kinase 5 (CDK5), mitogen‐activated protein kinase/extracellular signal‐regulated kinase (MAPK/ERK), and CaMKII are the major tau kinases associated with abnormal tau phosphorylation in the brain (Medeiros, Baglietto‐Vargas, & Laferla, 2011). We did not observe any significant differences in the total levels of these tau kinases (CDK5, ERK, and GSK3β) (Figure 4c). Notably, the results showed a significant reduction in the steady‐state levels of pGSK3β (phosphor‐GSK3β at residue Ser9) in the htau/STZ mice (Figure 4c). Together, our data indicate that the increase in tau hyperphosphorylation in htau/STZ mice is related to increases in the active form of GSK3β, as evidenced by reduction in phosphorylation.

### 2.6 Tau does not exacerbate the cognitive and synaptic deficits in T2DM mice

To assess the implication of tau on synaptic/cognitive deficits in T2DM, we used a genetic approach to ablate or overexpress tau levels in the db/db mice (Chen et al., 1996; Sharma, Elased, Garrett, & Lucot, 2010) (Figure S5A). Thus, we crossed the db/db mice with htau mice, generating db/db‐htau mice. db/db mice were also crossed with tauKO mice to generate db/db‐tauKO mice (Figure S5B).

**Figure 5:**
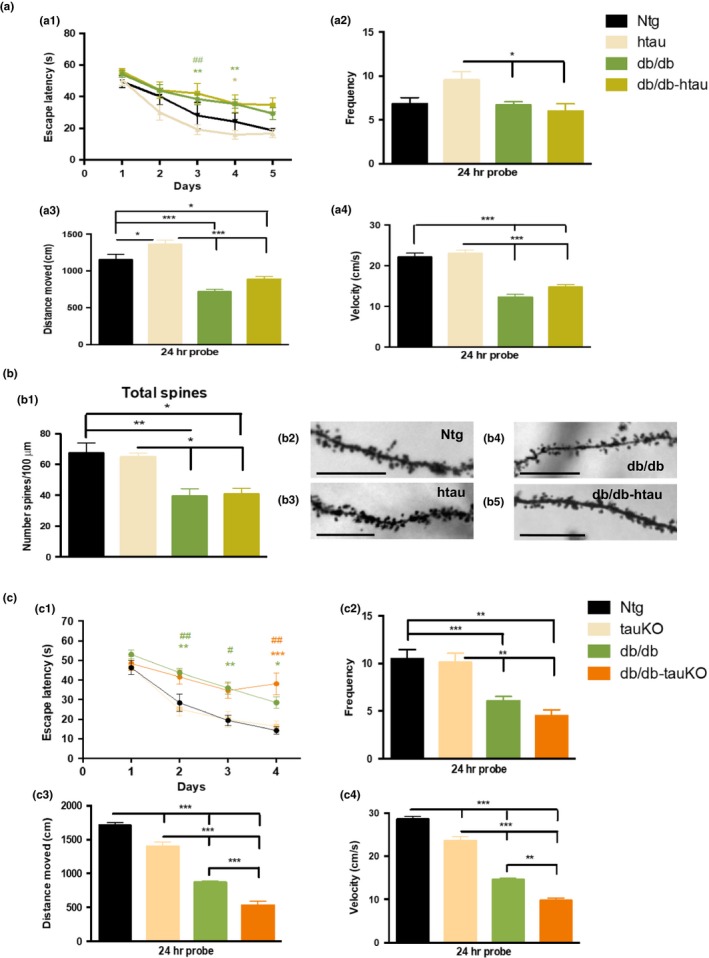
Tau does not exacerbate cognitive and synaptic deficits in type 2 diabetes db/db mice. (a) Ntg, htau, db/db, and db/db‐htau mice were trained on the spatial reference version of the MWM at 10 months of age. Acquisition curves (a1) are shown for the 5 days of training on the MWM. Two‐way ANOVA: trials [*F*(4, 155) = 28.56, *p* < 0.0001], treatment [*F*(3, 155) = 17.93, *p* < 0.0001], and interaction [*F*(12, 155) = 0.8314, *p* = 0.6180], Tukey's multiple comparisons test, **p < 0.01, *p < 0.05 (# significance vs. Ntg, green for db/db; * significance vs. htau, green for db/db, and yellow for db/db‐htau mice). (a2) Frequency of Ntg, htau, db/db, and db/db‐htau groups. Time to reach the platform is reduced in both db/db and db/db‐htau mice (one‐way ANOVA, **p = 0.0091, *F*(3, 37) = 4.456, Tukey's multiple comparisons test, *p < 0.05). Distance moved (a3) and velocity (a4) showed significant reduction for db/db and db/db‐htau mice compared to Ntg and htau groups (distance moved: one‐way ANOVA, ****p < 0.0001, *F*(3, 37) = 31.66, Tukey's multiple comparisons test, ***p < 0.001, *p < 0.05; velocity: one‐way ANOVA, ****p < 0.0001, *F*(3, 37) = 45.94, Tukey's multiple comparisons test, ***p < 0.001). *n* = 8–12 per group. (b) Dendritic spines analysis in Ntg, htau, db/db, and db/db‐htau mice. Stereological quantification (b1) showed significant decrease in total spines for both db/db (41.38%  7.34 vs. Ntg, and 39.35%  7.60 vs. htau) and db/db‐htau (39.38%  5.57 vs. Ntg, and 37.27%  5.77 vs. htau) mice compared to Ntg and htau mice (one‐way ANOVA, **p = 0.0013, *F*(3, 15)=8.797, Tukey's multiple comparisons test, **p < 0.01, *p < 0.05. *n* = 4–7 per group). (b2‐b5) Light microscopic images of radiatum layer in the hippocampal CA1 subfield in Ntg (b2), htau (b3), db/db (b4), and db/db‐htau (b5). (c) The ablation of tau does not recover the cognitive deficits in db/db mice. Ntg, tauKO, db/db, and db/db‐tauKO mice were trained on MWM at 8 weeks of age. Acquisition curves (c1) are shown for the 4 days of training. Two‐way ANOVA: trials [*F*(3, 120) = 47.94, *p* < 0.0001], treatment [*F*(3, 40) = 15.87, *p* < 0.0001], and interaction [*F*(9, 120) = 1.850, *p* = 0.0662], Bonferroni's multiple comparisons test, ***p < 0.001, **p < 0.01, *p < 0.05 (*significance vs. Ntg, and # significance vs. tauKO; green for db/db, and orange for db/db‐tauKO). Frequency (c2) of Ntg, tauKO, db/db, and db/db‐tauKO groups showed a significant reduction in both db/db and db/db‐tauKO mice (one‐way ANOVA, ****p < 0.0001, *F*(3, 39) = 10.35. Tukey's multiple comparisons test, ***p < 0.001, **p < 0.01). Distance moved (c3) and velocity (c4) showed significant reduction for db/db and db/db‐tauKO mice compared to Ntg and tauKO groups. Also significant differences were detected between Ntg and tauKO mice and between db/db and db/db‐tauKO (distance moved: one‐way ANOVA, ****p < 0.0001, *F*(3, 35) = 99.40, Tukey's multiple comparisons test, ***p < 0.001; velocity: one‐way ANOVA, ****p < 0.0001, *F*(3, 34) = 101.9, Tukey's multiple comparisons test, ***p < 0.001, **p < 0.01). *n* = 8–12 per group. The values represent means ± *SEM*. Scale bars: 10 µm

We found that db/db and db/db‐htau mice showed impairment in learning during MWM acquisition compared its respective control (Figure 5a1). However, no differences in learning were detected between db/db and db/db‐htau mice (Figure 5a1), indicating that tau does not exacerbate the cognitive deficits in the T2DM mice. Next, mice were tested 24 hr after the last training trial. Our findings showed that db/db and db/db‐htau mice displayed significant impairment on long‐term memory, as determined by a significant decrease in the frequency (Figure 5a2). No differences in memory were found between db/db and db/db‐htau mice (Figure 5a2). It is noteworthy that db/db and db/db‐htau mice displayed significant motor deficits that could be responsible for the low memory score in the MWM (Figure 5a3–a4).

Next, we evaluated dendritic spine density. Golgi staining and stereological analysis were performed in the stratum radiatum of CA1 hippocampal area. The quantification indicated that db/db and db/db‐htau mice displayed significant deficits in dendritic spine density compared to control mice (Ntg and htau mice) (Figure 5b), indicating that db/db mice have dendritic alterations that might affect cognitive function; however, the overexpression of wild‐type human tau and hyperphosphorylated tau accumulation (at residues Ser202/Thr205 stained by AT8 antibody) does not exacerbate these deficits (Figure S5A and S6).

To corroborate that tau does not contribute to cognitive/synaptic impairments in the T2DM mouse model, we ablated tau in the db/db mice by crossing them with tauKO mice generating the db/db‐tauKO mouse model. Mice were tested by the MWM test and we observed that both db/db and db/db‐tauKO mice displayed important deficits in learning during acquisition (Figure 5c1), while no differences in learning were detected between Ntg and tauKO mice. In addition, after 24 hr, db/db and db/db‐tauKO mice showed a significant decrease in the frequency (Figure 5c2). In agreement with the results described for db/db and db/db‐htau (Figure 5a3, a4), db/db and db/db‐tauKO mice displayed a reduction in both distance moved (Figure 5c3) and velocity (Figure 5c4) compared to Ntg and tauKO groups. Taken together, these data strongly suggest that db/db mice have tau‐independent cognitive impairment.

### 2.7 T2DM is associated with inflammation in the hippocampus

**Figure 6:**
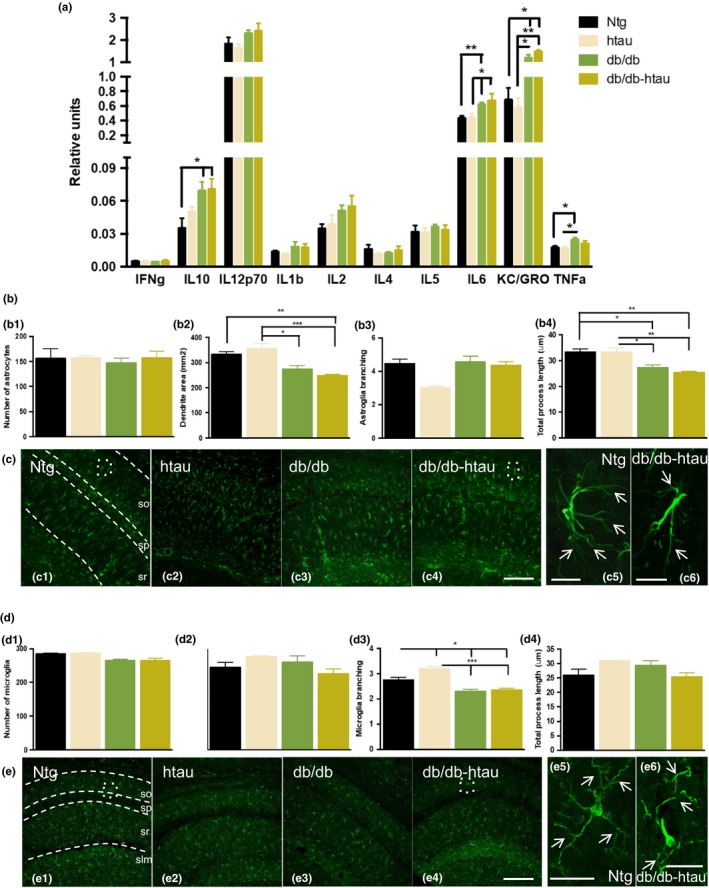
db/db mice displayed an exacerbate inflammatory response. (a) MSD cytokines analysis in Ntg, htau, db/db, and db/db‐htau mice revealed an inflammatory profile in db/db and db/db‐htau mice compared to age‐matched control with a significant increase in the levels of IL‐10, IL‐6, KC/GRO, and TNF‐α (IL‐10: one‐way ANOVA, *p = 0.0210, *F*(3, 17) = 4.224, Tukey's multiple comparisons test, *p < 0.05. IL‐6: one‐way ANOVA, **p = 0.0092, *F*(3, 14) = 5.698, Tukey's multiple comparisons tests, **p < 0.01, *p < 0.05. KC/GRO: one‐way ANOVA, **p = 0.0021, *F*(3, 14) = 8.253, Tukey's multiple comparisons test, **p < 0.01, *p < 0.05. TNF‐α: one‐way ANOVA, *p = 0.0316, *F*(3, 20) = 3.598, Tukey's multiple comparisons test, *p < 0.05. *n* = 5–8 per group). **(**b–c) Astroglia analysis of Ntg, htau, db/db, and db/db‐htau mice at 10 months of age. (b) Quantitative analysis of GFAP confocal images using Imaris software showed a reduction in astroglia dendritic area (b2) and total process length (b4) in db/db and db/db‐htau mice compared to age‐matched Ntg and htau (b2: one‐way ANOVA, ***p = 0.0005, *F*(3, 11) = 13.49. b4: one‐way ANOVA, ***p = 0.0008, *F*(3, 11) = 12.08. Tukey's multiple comparisons test, ***p < 0.001, **p < 0.01, *p < 0.05. *n* = 4 per group). (c) Representative confocal images for GFAP staining sections from Ntg (c1), db/db (c2), and db/db‐htau (c3) groups. Detail pictures for Ntg (c4) and db/db‐htau (c5) astroglial cells showing a reduction in the dendrite area and length (white arrows) in db/db‐htau mice. (d–e) Microglial analysis of Ntg, htau, db/db, and db/db‐htau mice at 10 months of age. (d) Quantitative analysis for confocal images taken from Iba‐1‐stained sections, using Imaris software, showing a decrease in microglia branching (d3) in db/db and db/db‐htau mice compared to Ntg and htau (one‐way ANOVA, ****p < 0.0001, *F*(3, 11) = 21.34, Tukey's multiple comparisons test, ***p < 0.001, *p < 0.05. *n* = 4 per group). (e) Representative confocal images for Iba1 stained sections from Ntg (e1), db/db (e2), and db/db‐htau (e3) groups. Detail pictures demonstrated a reduction in microglia branching (white arrows) in db/db‐htau (e5) compared to Ntg (e4) mice. The values represent means ± *SEM*. Scale bars: 500 µm (c1–c4, e1–e4), 250 µm (c5–c6), 25 µm (e5–e6). so: stratum oriens, sp: stratum pyramidale, sr: stratum radiatum, slm: stratum lacunosum‐moleculare

As we have demonstrated above, tau is not the main factor inducing cognitive/synaptic deficits in the T2DM mice, so we seek to investigate whether other factors that may be associated with these deficits. In regard with this idea, a growing body of evidence indicate that inflammatory signaling can impair memory and synaptic function (Donzis & Tronson, 2014). Therefore, we hypothesized that T2DM is accompanied by an increase in inflammation in the hippocampus, which might explain the cognitive/synaptic deficits described in T2DM mice. In order to address this question, we first analyzed the cytokines levels by ELISA. We observed a significant increase in interleukin (IL)‐10, IL‐6, KC/GRO, and TNF‐α in the hippocampus of db/db and db/db‐htau mice compared to control mice (Figure 6a).

Then, we analyzed the astroglial phenotype in Ntg, htau, db/db, and db/db‐htau mice (Figure 6b–c). The Imaris quantification of confocal images (Figure 6b) revealed a significant reduction in the astrocytes dendrite area (Figure 6b2) and the total process length (Figure 6b4) in the db/db and db/db‐htau mice. The high magnification confocal images corroborated the reduction in the dendritic area (white arrows, Figure 6c5 and c6) in the db/db‐htau mice compared to Ntg, as the ramification of astroglial processes is lower in the db/db‐htau mice (similar results were observed for the db/db mice, data no shown). Finally, we analyzed the microglia in those animals (Figure 6d–e). The Imaris quantification showed that db/db and db/db‐htau mice displayed a significant reduction in the microglia branching (Figure 6d3), as the microglial cells in the db/db‐htau mice displayed less process ramification compared to Ntg mice (white arrows, Figure 6e5,e6). No significant differences were observed in the number of astrocytes (Figure 6b1) or microglia (Figure 6d1). Thus, inflammation observed in these mice may be an important link between T2DM and AD and be associated with the cognitive/synaptic impairment in T2DM.

## 3 DISCUSSION

Clinical data indicate that DM induces significant neuronal dysfunction and cognitive impairment in aging populations (Biessels & Reagan, 2015). However, the underlying cellular/molecular mechanisms linking DM to cognitive dysfunction remain unknown. Recent evidence has shown that DM induces tau pathology, which has a major role in synaptic‐related processes (Baglietto‐Vargas, Shi, Yaeger, Ager, & LaFerla, 2016; Qu et al., 2011; Sutherland et al., 2017). Therefore, we investigated whether tau is a key molecular driver of DM‐induced cognitive/synaptic deficits. Our study provides compelling evidence that tau differentially contributes to the cognitive/synaptic impairments induced by DM. On one hand, tau is critical for T1DM‐like disease to induce profound synaptic and cognitive deficits. On the other hand, the presence or absence of tau did not alter the cognitive performance in a T2DM mouse model.

Tau has an important role in stabilizing neuronal microtubules and in regulating axonal transport (Wei et al., 2015). New evidences have demonstrated that tau also regulates important processes related to synaptic function, as tau can be detected in dendrites and pre‐ and postsynaptic components of healthy neurons (Regan, Whitcomb, & Cho, 2017). In pathological conditions, such as AD, tau alterations are associated with synapse dysfunction, neuronal degeneration, and cognitive decline (Alonso, Zaidi, Grundke‐Iqbal, & Iqbal, 1994). Here, we demonstrate that T1DM induces cognitive impairments in mice that overexpress wild‐type human tau. These findings are in agreement with previous data from our group, which found that genetic ablation of tau mitigated behavioral deficits under T1DM conditions (Abbondante et al., 2014). Overall, our findings suggest that tau is a critical mediator of the cognitive impairments associated with T1DM.

Synaptic dysfunction is thought to be an early and important pathogenic step in both DM and AD (Regan et al., 2017). The majority of excitatory synaptic processes occur in dendritic spines (Rochefort & Konnerth, 2012), and synaptic function is intimately related to structural and morphological changes in spines. Emerging evidences suggest that these alterations are associated with the symptomatology of many neurological disorders (Kasai, Fukuda, Watanabe, Hayashi‐Takagi, & Noguchi, 2010). Our results demonstrate that htau mice, under T1DM conditions, show significant reductions both in the total number of dendritic spines and in mushroom dendritic spines in the hippocampus. These findings suggest that tau is necessary for T1DM to induce defects in synaptic processing and neuronal communication in a key region of the brain, necessary for normal learning and memory function. Additionally, our study also demonstrates that presynaptic areas are significantly affected in htau/STZ mice.

We next investigated the pathological cascade responsible for the tau‐dependent synaptic deficits in T1DM. The nonreceptor tyrosine kinase Src, Fyn, is an important modulator of long‐term potentiation (LTP) induction and regulates N‐methyl‐D‐aspartate receptor (NMDAR) transmission (Nygaard, 2017). Activated Fyn phosphorylates the NR2A and NR2B subunits of NMDAR, resulting in increased synaptic expression and enhanced receptor transmission (Trepanier et al., 2012). Enhanced dendritic tau may serve as a protein scaffold to deliver more Fyn to the postsynaptic sites, which then phosphorylate subunit 2 of the NMDAR (GluN2B) to stabilize a greater proportion of interactions between NMDARs and postsynaptic density 95 (PSD95) (Mondragón‐Rodríguez et al., 2012; Trepanier et al., 2012). This occurrence could boost an overactivation of NMDARs during excitatory glutamate neurotransmission, resulting in excitotoxic effects on the neurons (Mondragón‐Rodríguez et al., 2012). Herein, we demonstrated a decrease in phosphor‐Fyn. Fyn contains an autoinhibitory phosphorylation site Tyr527 (Tyr530 in human Fyn) (Boggon & Eck, 2004), so the phosphorylation of Fyn leads to its inhibition. Therefore, the reduction in phosphor‐Fyn could lead to its activation and the excitotoxic effects described above. It has been also proposed that Fyn phosphorylates tau, and that this interaction could affect AD pathogenesis (Lee et al., 2004), and contribute to the increase in tau phosphorylation observed in this study.

Calcium dysregulation is another potential downstream mechanism of tau (Forner et al., 2017). For example, calcium levels are increased in the brains of AD patients. Intracellular calcium and calcium‐dependent protease levels are higher in neurons containing NFTs, and the level of calcium/calmodulin‐dependent protein kinase II is elevated in neurodegenerative neurons (McKee, Kosik, Kennedy, & Kowall, 1990). CaMKII is a complex protein kinase, known to have a fundamental role in synaptic plasticity and memory formation, and has also been suggested to be a tau kinase (Ghosh & Giese, 2015). CaMKII is regulated by autophosphorylation (Wei et al., 2015) and our study has shown an increase in phosphor‐CaMKII, indicating an increase in its activity, which may contribute to the tau hyperphosphorylation observed in htau mice under diabetic conditions.

It has been demonstrated that diabetes promotes aberrant tau modifications through insulin signaling in humans and animal models, and the prevalent hypothesis is that hyperphosphorylation, and misfolding and fibrillization of tau impair synaptic plasticity (Qu et al., 2011; Wang & Mandelkow, 2015). Therefore, we next investigated the effect of T1DM on tau phosphorylation. We identified a marked increase in tau phosphorylation at residues Ser202/Thr205 (AT8) in hippocampal synaptosomes from T1DM induced htau mice and also confirmed by immunohistochemistry. These findings are in agreement with previous studies that found increased tau phosphorylation in mouse models of T1DM and T2DM (Guo et al., 2016; Kim, Backus, Oh, Hayes, & Feldman, 2009), in diabetic monkeys (Morales‐Corraliza et al., 2016), and in *postmortem* brain samples from patients with T2DM (Liu, Liu, Grundke‐Iqbal, Iqbal, & Gong, 2009).

Insulin regulates tau phosphorylation in vitro (Hong & Lee, 1997) and in vivo (Schubert et al., 2003). Thus, impaired insulin signaling could increase tau phosphorylation and cleavage (Kim et al., 2009). Under normal conditions, insulin signaling, via the insulin receptor, leads to GSK3β inactivation, whereas insulin resistance drives GSK3β activation leading to an increase in phosphorylated tau (Clodfelder‐Miller, Zmijewska, Johnson, & Jope, 2006). Here, we found a significant activation of GSK3β in htau/STZ mice, as levels of phosphor‐GSK3β were reduced. These results are in concordance with previous data from our research group and others (Dey, Hao, Wosiski‐Kuhn, & Stranahan, 2017), where we showed that T1DM led to the activation of GSK3β and tau hyperphosphorylation (Abbondante et al., 2014). We have also evaluated other main tau kinases such as CDK5, ERK, and CaMKII implicated in abnormal tau phosphorylation in the brain (Mondragón‐Rodríguez et al., 2012). As we have described above, we observed an increase in phosphor‐CaMKII, which correspond with its activated form and may also contribute to tau pathology (Guo et al., 2017).

T2DM is also associated with cognitive impairment (Stolk et al., 1997). Here, we investigated whether this impairment is dependent on tau. Our data showed that reducing or increasing tau levels did not alter the synaptic/cognitive performance of T2DM mice, indicating that tau did not play a central role in the cognitive/synaptic deficits found in T2DM, even though we have observed an increase in tau phosphorylation in db/db‐htau mice compared to htau mice. It is important to mention that tauKO mice did not show behavioral and synaptic deficits despite being null for the tau gene, as previously has been described (Abbondante et al., 2014). The lack of tau has been reported to be related to a significant increase in the microtubule‐associated protein 1A (MAP1A) and this increase may compensate the loss of tau at younger ages (Ke et al., 2012). Only in aged mice, in which MAP1A is not upregulated, tauKO mice develop behavioral impairments (Ke et al., 2012). It has also been described that the ablation of tau improves mitochondrial function and cognitive abilities in tau^–/–^ mice at 3 months of age (Jara, Aránguiz, Cerpa, Tapia‐Rojas, & Quintanilla, 2018). Therefore, the discordance observed with our results may be due to the different age of the analysis.

Next, we investigated other causative factor such as inflammatory process, mitochondrial dysfunction, or oxidative stress that may have a more critical role in the synaptic and cognitive deficits in T2DM (Carvalho et al., 2013; Chornenkyy, Wang, Wei, & Nelson, 2018; Ling et al., 2018; Pintana et al., 2012; Tumminia, Vinciguerra, Parisi, & Frittitta, 2018; Verdile et al., 2015). In addition, others authors have shown that db/db mice have reductions in brain weight and spine density, which were worsened in APP/PS1xdb/db mice, indicating a role for Aβ in these deficits (Infante‐Garcia, Ramos‐Rodriguez, Galindo‐Gonzalez, & Garcia‐Alloza, 2016). Moreover, a new disease mechanism involving the interaction of misfolded proteins (Aβ in AD and islet amyloid polypeptide in T2DM) through cross‐seeding has been proposed to explain the interaction between both diseases (Moreno‐Gonzalez et al., 2017). Here, we examined whether the inflammatory process is a possible pathological mechanism driving the cognitive deficits in db/db mice.

As in AD, T2DM has been characterized as a chronic, subacute inflammatory state (McGeer & McGeer, 1999). There is substantial evidence that inflammation is more than a peripheral factor in AD, and is likely that inflammatory processes play a crucial role in T2DM and AD pathogenesis (Donzis & Tronson, 2014; Rao, Kellom, Kim, Rapoport, & Reese, 2012). We have observed important changes in astroglial and microglial cells in db/db and db/db‐htau mice compared to Ntg or htau mice. With this regard, astroglial cells displayed a reduction in the dendritic area and the total process length, and microglia cells had reduced branching in T2DM mice. These changes are in agreement with an activated glial cell phenotype (Streit, Xue, Tischer, & Bechmann, 2014). Moreover, obesity‐related metabolic disorders, such as T2DM and insulin resistance, produce a chronic state of low‐grade systemic inflammation leading to the overproduction of pro‐inflammatory cytokines (Fishel et al., 2005). The elevated synthesis of pro‐inflammatory cytokines, such as IL‐6, characterizes the early or preclinical stages of T2DM (Badawi et al., 2010). These inflammatory mediators can activate brain‐resident microglia and astrocytes, leading to CNS inflammation and synaptic dysfunction (Donzis & Tronson, 2014; Rao et al., 2012). In our study, we analyzed several inflammatory cytokines in the hippocampus of Ntg, htau, db/db, and db/db‐htau mice, and observed significant increases in IL‐10, IL‐6, KC/GRO, and TNF‐α in T2DM mice. Therefore, chronic inflammation may represent an underlying mechanism common to AD and metabolic disorders (Ribe & Lovestone, 2016), and the pro‐inflammatory response in T2DM mice could account for the cognitive and synaptic deficits common in the model.

Although these findings provide important evidence of the role of tau in mediating the cognitive and synaptic impairments found in type 1 and type 2 diabetes, it is important to consider the current limitations of existing animal models and the limitations of using a pharmacological approach to model a human disease in rodents. In sum, our study demonstrates that the protein tau is a crucial downstream target of the insulin pathway, and mediates the cognitive deficits observed in T1DM mouse models, whereas in T2DM mice, other factors such as chronic inflammatory may be responsible for cognitive deficits. Overall, our data demonstrate a critical differential role of tau in the synaptic and cognitive deficits associated with T1DM and T2DM. Therefore, our results provide new insights into the mechanisms by which DM induces cognitive decline, and may open new avenues for targeting different neurological disorders associated with the diabetic condition.

## 4 EXPERIMENTAL PROCEDURES

### 4.1 Human samples

The superior frontal gyrus (SFG) from human autopsy specimens was provided by the University of California Alzheimer's Disease Research Center (UCI‐ADRC) and the Institute for Memory Impairments and Neurological Disorders. The utilization of *postmortem* human samples was approved by the corresponding biobank ethics committees. The cases selection was made in base to their clinical records about diabetic conditions. All cases were scored for Braak tau pathology (Braak & Braak, 1991). Table 1 summarizes the demographics of the human samples used.

**Table 1:** Human sample information

AD (*n* = 5)						AD + DM (*n* = 5)					
Case year	Case no.	Age	Sex	PMI (hr)	Tangle stage	Case year	Case no.	Age	Sex	PMI (hr)	Tangle Stage
2007	7	87	M	3.33	5	2011	6	83	M	3.42	5
2008	13	89	F	3.67	5	2012	20	90+	F	5	4
2002	8	84	M	5.3	5	2013	27	80	M	3.75	5
2012	3	81	M	4.17	4	2015	8	79	M	3.92	4
2003	11	86	M	5.5	5	2015	47	82	M	8.83	6

Table summarizes human sample information, including the year and number of the case, age, sex, postmortem interval (PMI), and tangle stage.

### 4.2 Transgenic mice

For type 1 diabetes, 15‐month‐old homozygous htau and Ntg male and female mice with the same genetic background (C57BL6N) were used. To induce type 1 diabetes, the mice received two injections of STZ (75 mg/kg, i.p.) diluted in 0.1 mol/L citrate buffer (pH 4.5) at 14 months of age (Ke, Delerue, Gladbach, Gotz, & Ittner, 2009; Qu et al., 2011) (Figure S7A).

For type 2 diabetes, we used the male and female db/db mice (Chen et al., 1996). We crossed the db/db mice with the htau mice, generating the hemizygous db/db‐htau mice (Figure S5A), and with the tauKO mice (Abbondante et al., 2014) generating homozygous db/db‐tauKO mice (Figure S5B). Ntg, htau, db/db, and db/db‐htau male and female mice were analyzed at 10 months old (Figure S7B), and Ntg, tauKO, db/db, and db/db‐tauKO male and female mice were analyzed at 8 weeks old (Sharma et al., 2010) (Figure S7C).

All animal procedures were performed in accordance with NIH and University of California guidelines and Use Committee at the University of California, Irvine. A detailed description of the transgenic mice is included as supplemental data.

### 4.3 Methods

A detailed description of the methods used in the current manuscript, including weight, blood glucose and insulin measurements, behavioral test, tissue preparation, Golgi stain, spine analysis, synaptosome extracts, immunoblotting, immunohistochemistry, quantitative analyses, pro‐inflammatory ELISA, and statistical analyses, is added as supplemental data (Baglietto‐Vargas et al., 2015; Clodfelder‐Miller et al., 2006; Franklin & Paxinos, 2008; Sanchez‐Varo et al., 2012; Sandler & Mcdonnell, 2016).

## CONFLICT OF INTEREST

None declared.

## AUTHOR CONTRIBUTIONS

L.T.E., D.B.V., and F.M.L. conceived and designed the experiments. L.T.E., C.N., C.C., L.C., S.F., and A.C.M. performed the experiments. L.T.E., R.R.A., D.B.V., and F.M.L. analyzed the data. L.T.E., R.R.A., G.A.P., C.W.C., D.B.V., and F.M.L. contributed to the writing of the manuscript.
