# Supplementary figures and images for: Tau underlies synaptic and cognitive deficits for type 1, but not type 2 diabetes mouse models
Source: Aging Cell. 2019 Feb 27;18(3):e12919. doi: 10.1111/acel.12919 (PMC6516168; doi:10.1111/acel.12919)

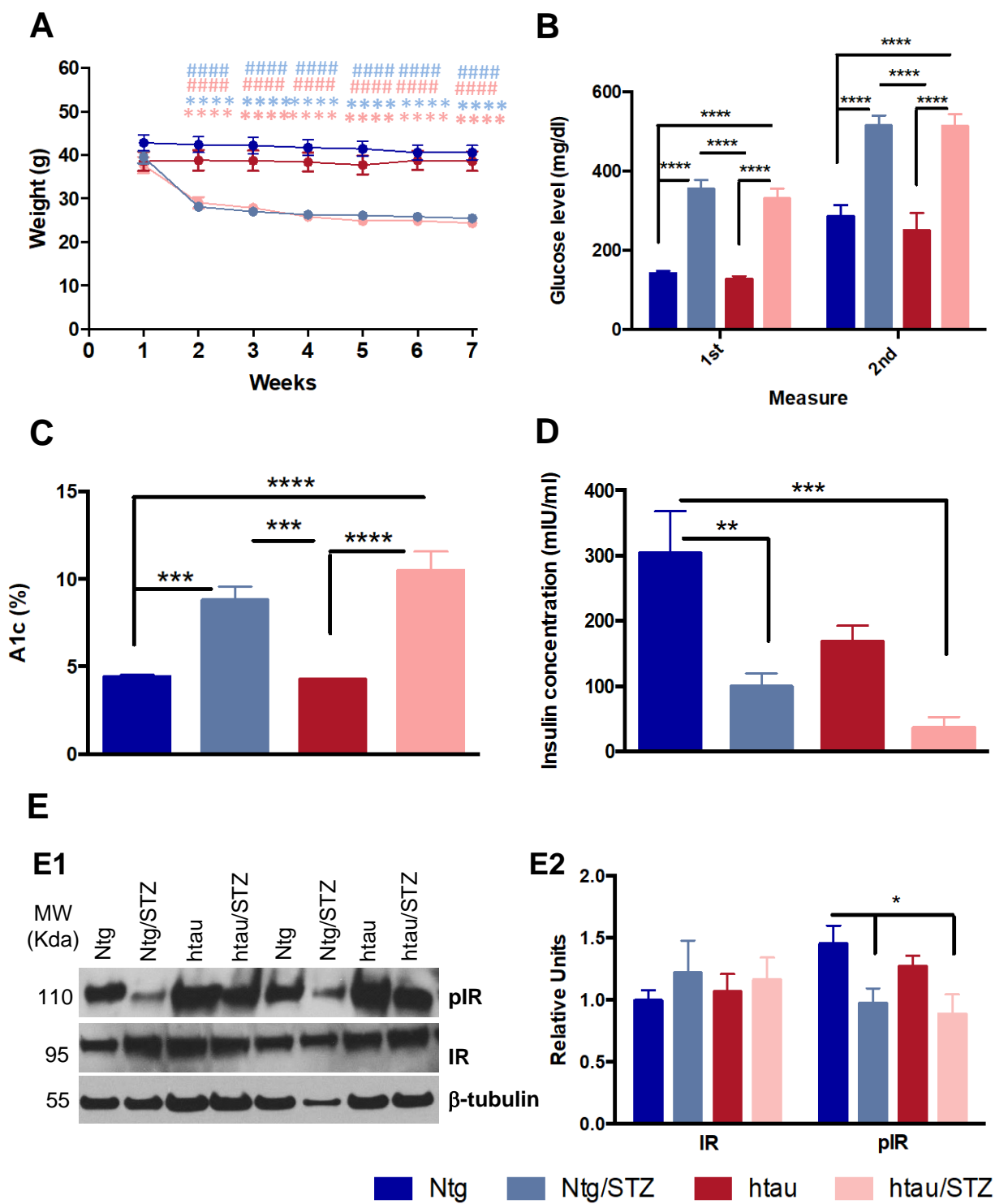

**Figure S1**

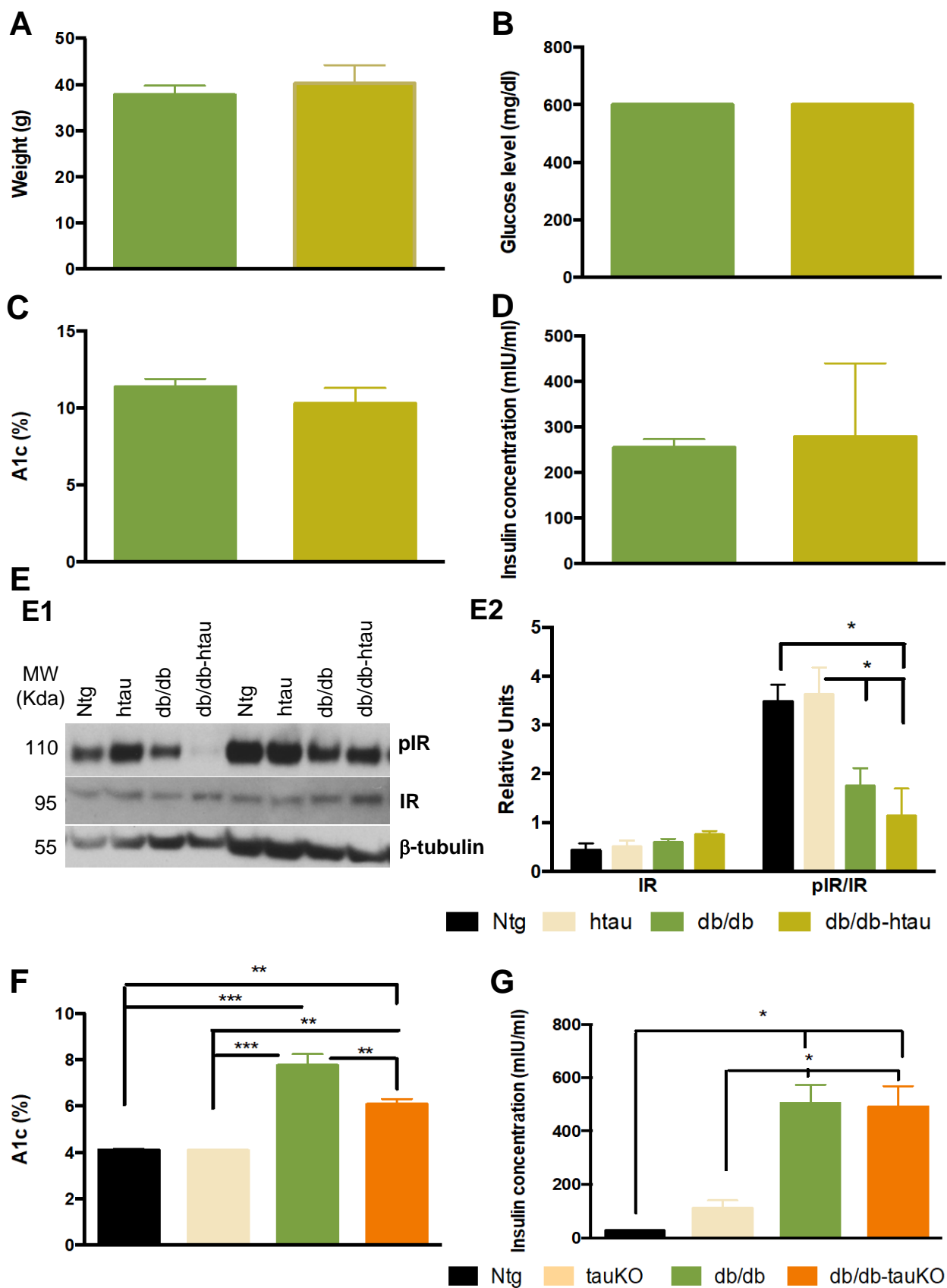

**Figure S2**

A

A1

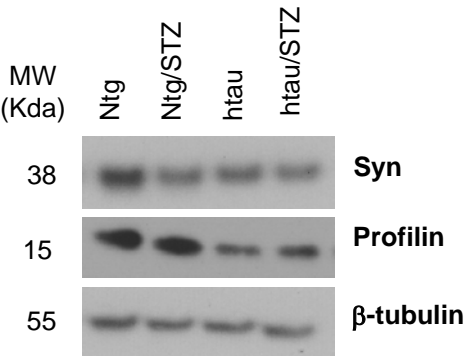

A2

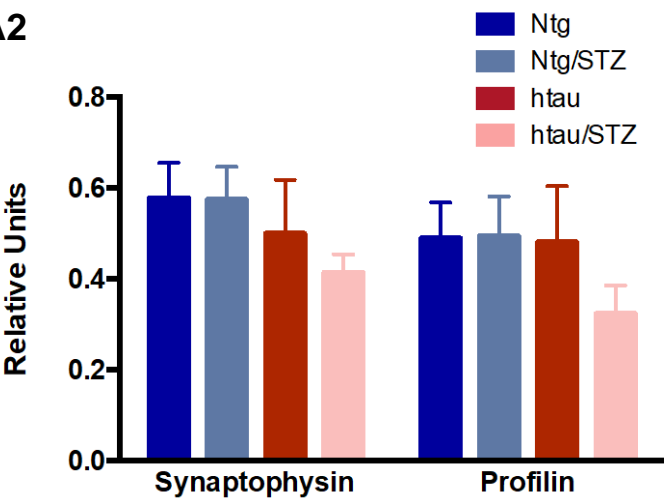

Figure S3

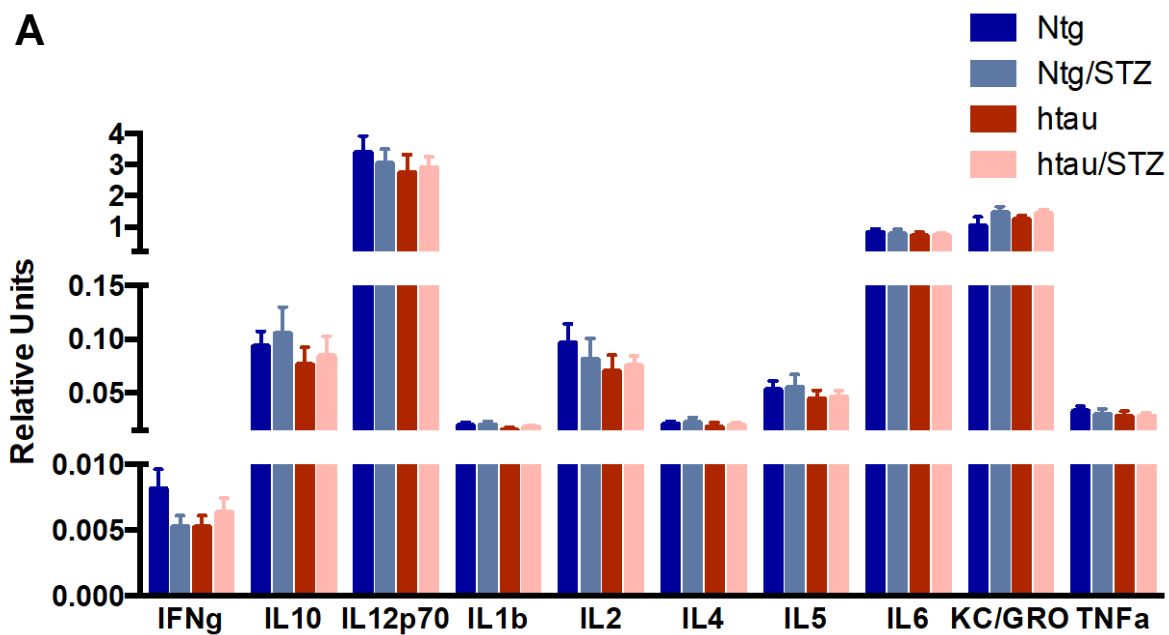

**Figure S4**

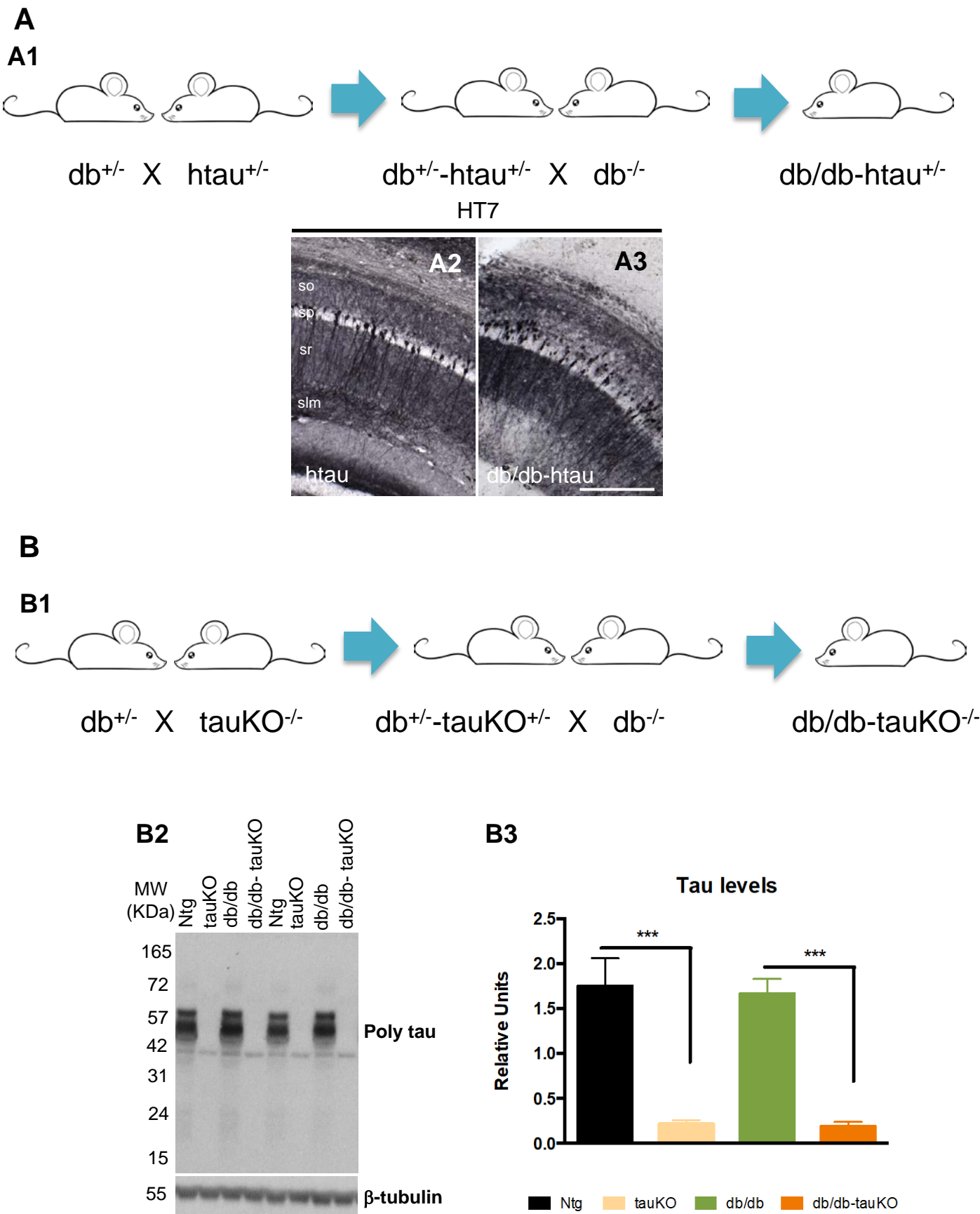

**Figure S5**

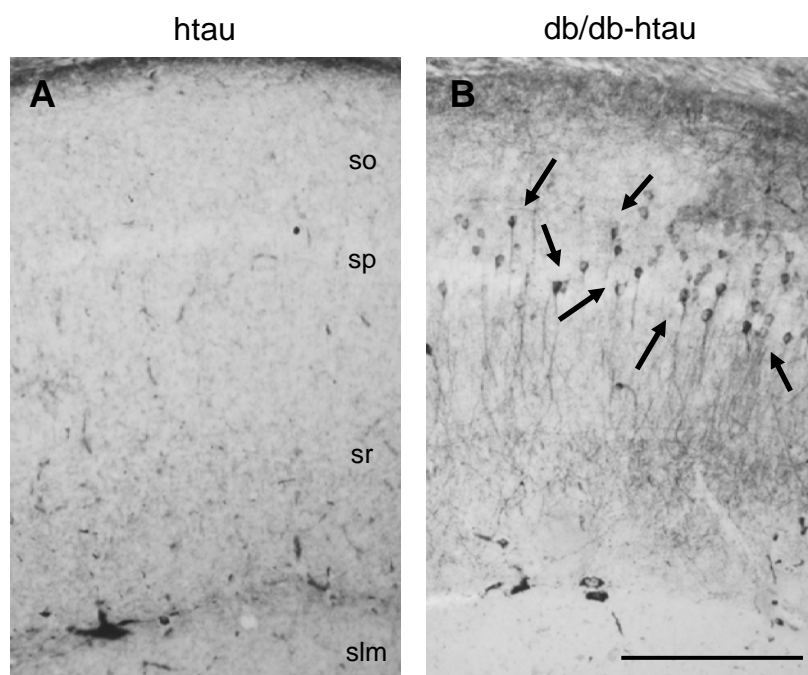

**Figure S6**

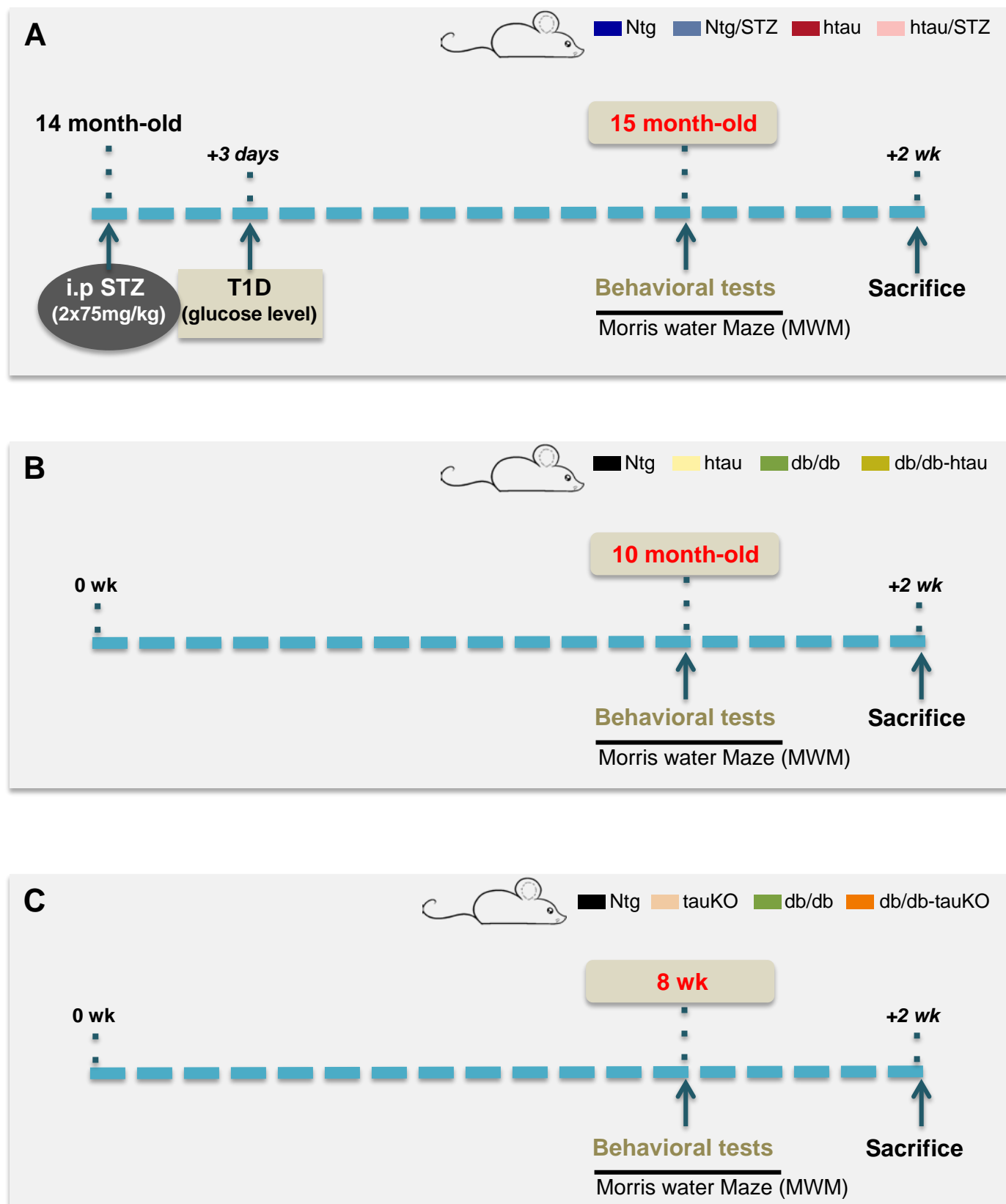

**Figure S7**

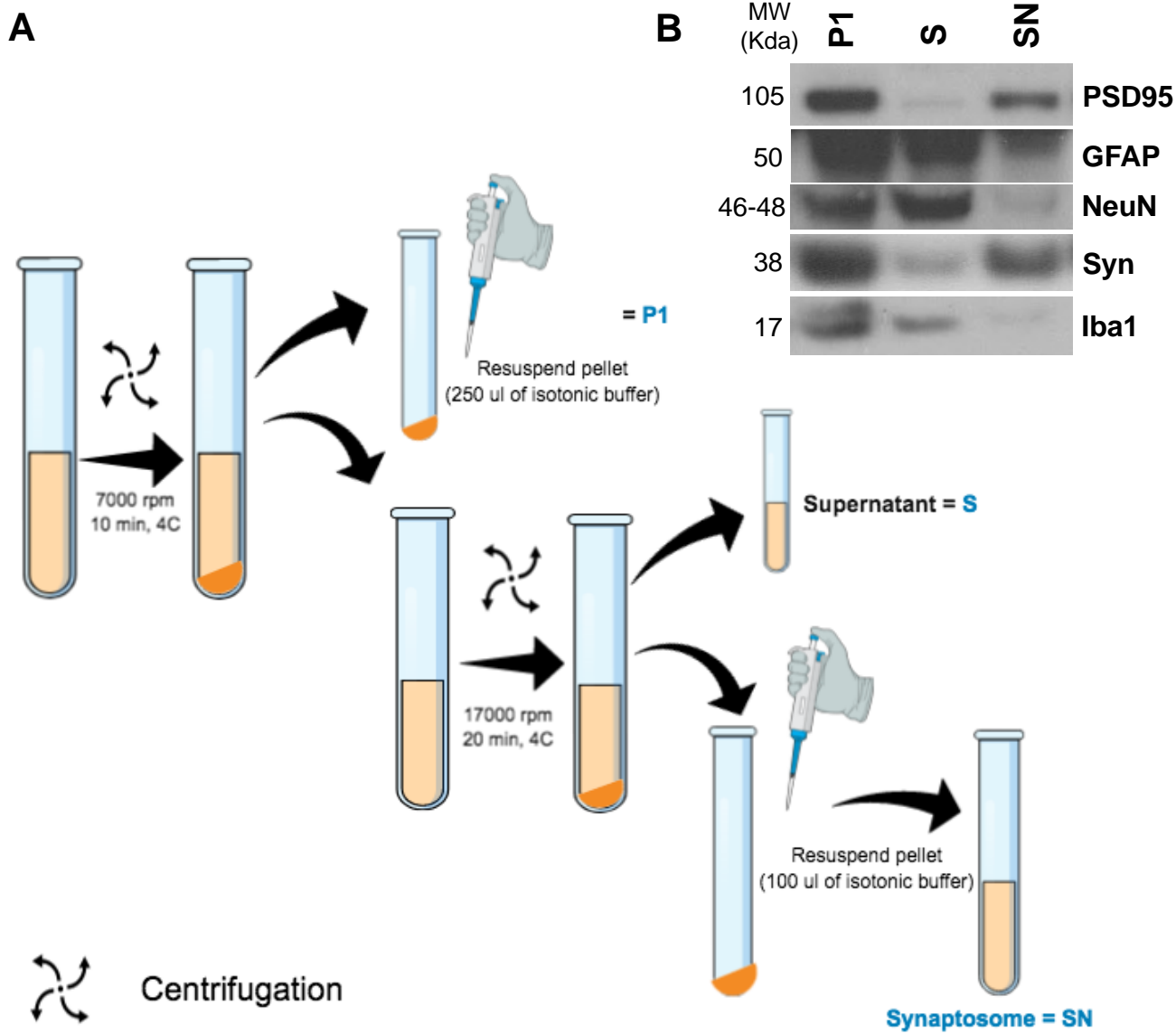

**Figure S8**

Supplement: Supplementary file 1 [file ACEL-18-e12919-s001.pdf]
