# Supplementary material for: Tau underlies synaptic and cognitive deficits for type 1, but not type 2 diabetes mouse models
Source: Aging Cell. 2019 Feb 27;18(3):e12919. doi: 10.1111/acel.12919 (PMC6516168; doi:10.1111/acel.12919)
Supplement: Supplementary file 2 [file ACEL-18-e12919-s002.docx]

**Supporting information**

**Supplemental references**

Baglietto-Vargas, D., Chen, Y., Suh, D., Ager, R. R., Rodriguez-Ortiz, C. J., Medeiros, R., … LaFerla, F. M. (2015). Short-term modern life-like stress exacerbates A??-pathology and synapse loss in 3xTg-AD mice. *Journal of Neurochemistry*, *134*(5), 915–926. <https://doi.org/10.1111/jnc.13195>

Franklin, K. B. J., & Paxinos, G. (2008). *The mouse brain in stereotaxic coordinates*. Boston.

Sanchez-Varo, R., Trujillo-Estrada, L., Sanchez-Mejias, E., Torres, M., Baglietto-Vargas, D., Moreno-Gonzalez, I., … Gutierrez, A. (2012). Abnormal accumulation of autophagic vesicles correlates with axonal and synaptic pathology in young Alzheimer’s mice hippocampus. *Acta Neuropathologica*, *123*(1), 53–70. https://doi.org/10.1007/s00401-011-0896-x

SANDLER, C. N., & MCDONNELL, M. E. (2016). The role of hemoglobin A1c in the assessment of diabetes and cardiovascular risk. *Cleveland Clinic Journal of Medicine*, *83*(suppl 1), S4–S10. https://doi.org/10.3949/ccjm.83.s1.02

**Supplemental experimental procedures**

**Transgenic mice**

For type 1 diabetes, 15 months old homozygous wild type human tau and Non-transgenic mice were used. All mice (Ntg and htau) had the same genetic background (C57BL6N background). This htau expresses the full-length human MAPT containing exons 2 and 3 (2N) and exon 10 (4R). To induce type 1 diabetes in Ntg and htau mice, the mice received two injections of STZ (75 mg/kg, i.p.) diluted in 0.1 mol/L citrate buffer (pH 4.5) at 14 months of age. STZ, a glucosamine-nitrosourea compound, is toxic to the insulin-producing β-cells of the pancreas, and i.p. administration of STZ induces hyperglycemia and insulin deficiency, rendering it a valuable model to study T1DM (Y D Ke et al., 2009; Qu et al., 2011). 14 months old Ntg and htau control mice (without STZ treatment) received two injections of vehicle solution. The onset of diabetes was confirmed 3 days after STZ treatment by assessing glucose levels (Nipro Diagnostics, Fort Lauderdale, FL) in blood samples collected from the tail. After a month, the animals underwent behavioral tests (Figure S7A).

For type 2 diabetes, we used the db/db mice, a well-established T2DM model displaying obesity, hyperglycemia, insulin-resistance and hyperinsulinemia (H. Chen et al., 1996). To determine whether tau is essential for the deficits induced by T2DM, we used a genetic gain-of-function by crossing the db/db mice with the htau mice, generating the hemizygous db/db-htau mice (Figure S5A). We also used a genetic approach to ablate tau levels. We crossed the db/db mice to tauKO mice (Abbondante et al., 2014) and we generated homozygous db/db-tauKO mice (Figure S5B). Ntg, htau, db/db and db/db-htau mice were analyzed at 10 months old, since db/db lifespan is 10 to 12 month of age (Figure S7B). For the Ntg, tauKO, db/db and db/db-tauKO mice, the analysis was performed at 8 weeks old, as db/db mice manifest cognitive and synaptic deficits as early as 8 weeks (Sharma et al., 2010) (Figure S7C).

All animal procedures were performed in accordance with NIH and University of California guidelines and Use Committee at the University of California, Irvine.

**Weight, blood glucose and insulin measurements**

The animals for the T1DM study were weighted weekly. The onset of diabetes was confirmed 3 days after STZ treatment by assessing glucose levels (Nipro Diagnostics, For Laurderdale, FL) in blood samples collected from the tail (Figure S7A) of fasting animals (6 hours of fasting). Only animals with blood glucose levels ≥200 mg/dL were used in the experiments (Clodfelder-Miller et al., 2006). Blood glucose level and A1c were determined immediately before euthanizing all groups (type 1 and type 2 diabetes) in fasting animals. A1c is a measurement of glycated hemoglobin (HbA_1c_) and represents the most widely accepted indicator of long-term glycemic exposure (SANDLER & MCDONNELL, 2016). In addition, insulin levels were also measured before T1DM and T2DM studies by ELISA (mouse insulin ELISA kit, Thermo Fisher Scientific, Rockford, IL, USA).

**Behavioral Test: Morris water maze**

Mice were trained to swim to a 14-cm diameter circular Plexiglas platform submerged 1.5 cm beneath the surface of the water and invisible to the mice while swimming. The platform was located in a fixed position, equidistant from the center and the wall of the tank. Mice were subjected to four training trials per day. During each trial, mice were placed into the tank at one of four designated start points per day in a pseudorandom order. Mice were trained for as many days as needed to reach the training criteria of 25 seconds (escape latency). If the mice failed to find the platform within 60 seconds, they were manually guided to the platform and allowed to remain there for 5 seconds. The probe trial was assessed 24 hours after the last training session and consisted of a 60-second free swim in the pool without the platform. Performance was monitored with the EthoVision XT video tracking system (Noldus Information Technology, Leesburg, VA).

**Tissue preparation**

After euthanasia, the animals were perfused transcardially with 0.1M phosphate-buffered saline (PBS, pH7.4). Next, hippocampus was used to collect synaptosomes (Sanchez-Varo et al., 2012), to stain with Golgi solution (Baglietto-Vargas et al., 2015), for MSD analysis or for immunohistological stain.

**Golgi Stain**

Mice were perfused transcardially with 0.1M phosphate-buffered saline (PBS, pH 7.4) and brains were processed using superGolgi Kit (Bioenno Tech LLC, Santa Ana, CA), as described previously (Baglietto-Vargas et al., 2015). Brains were incubated for 11 days in impregnation solutions, followed by 2 days incubation in a post-impregnation solution. Once the impregnation of neurons was complete, thick (150 μm) free-floating sections were obtained using a HA752 vibratome (Campden Instruments Ltd, Lafayette, IN) and serially collected in mounting buffer. Sections mounted on coated slides were stained and post-stained respectively for 20 min, dehydrated in graded ethanol, cleared with xylene and coverslipped with DPX (VWR, Visalia, CA, USA) mounting medium.

**Dendritic and Spine Analysis**

Stereological quantifications were performed using Neurolucida software from Microbrightfield Bioscience (MBF Bioscience, Williston, VT, USA) to determine the number of spines in the stratum radiatum of the hippocampal CA1 region. Briefly, every 2nd section was used through the entire antero-posterior extent of the hippocampus (between -1.46 mm anterior and -3.40 mm posterior to Bregma according to the atlas of Franklin and Paxinos) (Franklin & Paxinos, 2008). In CA1 region, sr was defined using a 5x objective and spines were counted using a 100x/1.4 objective. The coefficients of error (CEs) value for each individual animal ranged between 0.03 and 0.08. Dendritic spine length was traced using a 100x/1.4 objective and data were analysed via Neurolucida Explorer software. For dendritic morphological analysis, 6 dendrites per section (5 sections per animal, n=6 animals per group) in CA1 hippocampal area were traced using Neurolucida software and evaluated. Morphologically, for the quantification, spines were divided into 3 subtypes: mushroom, stubby and thin spines.

**Synaptosome extracts**

Briefly, the tissue (the hippocampus from one mouse and the superior frontal gyrus from human patients) was homogenized (using a Dounce homogenizer) in DEPC-treated water (Ambion, Thermo Fisher Scientific) supplemented with 0.32 M sucrose, 20 mM Tris-HCl, 0.5 M EDTA and 0.5 M EGTA (pH 7.4), containing complete protease and phosphatase inhibitor cocktails (Sigma-Aldrich, San Luis, MO, USA). After homogenization, the crude synaptosomal fraction (synaptosomes plus mitochondria) was isolated by two sequential centrifugations (1,500xg, 10 min followed by 12,500xg, 20 min; at 4°C). The protein content of the synaptosomal fractions was determined using the Bradford assay. For Western blots experiments synaptosomal preparations were temporally stored at -80^0^ C. Western-blot analyses demonstrated that the synaptosome extract are rich in synaptic markers and nuclear neuronal or glial markers are not presented (Figure S8).

**Immunoblotting**

Equal amounts of protein (5 μg) were separated on 10% Bis-Tris gel (Invitrogen, Carlsbad, CA), and transferred to nitrocellulose membranes. Membranes were blocked for 1 hour in 5% (w/v) suspension of Bovine Serum Albumin (BSA; Gemini Bio-Products, West Sacramento, CA, USA) in 0.2% Tween 20 Tris-buffered saline (pH 7.5). After blocking, the membranes were incubated overnight at 4°C, with one of the following primary antibodies: anti-GluR1 (1:1000; Millipore, Burlington, MA), anti-*p*-GluR1 (Ser845; 1:1000; Millipore), anti-CamKII (1:1000; Cell Signaling ), anti-*p*-CamKII (1:1000; Cell Signaling), anti-Fyn (1:1000; Cell Signaling), anti-*p*-Fyn (phospho Y530; 1:1000; Abcam, Cambridge, MA, USA), anti-AT8 (1:1000; Thermo Scientific), anti-AT180 (1:1000; Thermo Scientific), anti-AT270 (1:1000; Thermo Scientific), anti-p-tau paired helical filament (PHF; 1:1000; Dr. Peter Davies, Albert Einstein College of Medicine, Manhasset, NY), anti-HT7 (1:1000; Thermo Scientific), anti-CDK5 (1:1000; Millipore), anti-GSK3β (1:1000; Cell Signaling), anti-*p*-GSK3β (Ser9; 1:1000; Cell Signaling), anti-extracellular signal-regulated kinase (ERK; 1:500; Cell Signaling), anti-*p*-ERK (1:500; Cell Signaling), anti-postsynaptic density protein 95 (PSD95; 1:1000; Cell Signaling); anti-synaptophysin (1:2000; Abcam), anti-Poly tau (1:3000; Dako), anti-GFAP (1:1000; Abcam), anti-NeuN (1:1000; Cell Signaling), anti-Iba1 (1:2000; Abcam), anti-BDNF (1:500; Abcam), anti-profilin (1:1000; Cell Signaling), anti-insulin receptor (IR; 1:1000; Millipore), anti-*p*-IR (Tyr972; 1:1000; Millipore); anti-β-tubulin (1:2000; Cell Signaling). The membranes were washed in tween-TBS for 20 min and incubated at 20°C with the specific secondary antibody at a dilution of 1:10000 (Pierce Biotechnology, Waltham, MA, USA) for 60 min. The blots were developed using Super Signal (Thermo Fisher Scientific).

**Immunohistochemistry**

Coronal free-floating sections (40μm thick) were pretreated with 3% H2O2/3% methanol in Tris-buffered saline (TBS) for 30 min to block endogenous peroxide activity. After TBS wash, sections were incubated first in TBS with 0.1% Triton X-100 (TBST) for 15 min, and then in TBST with 2% bovine serum albumin (BSA, Sigma-Aldrich) for 30 min. Sections were incubated with anti-synaptophysin (1:1000; Abcam), anti-HT7 (1:500; Thermo Scientific), anti-AT8 (1:500; Thermo Scientific), anti-GFAP (1:5000; Abcam) and anti-Iba1 (1:500; Abcam) in TBS + 5% normal horse serum overnight at room temperature. Sections were then incubated with biotinylated anti-mouse, 1:500 in TBS + 2%BSA + 5% normal serum for 1hr at 20^0^C, followed by Vector ABC Kit and DAB reagents (Vector Laboratories, Burlingame, CA, USA) to visualize staining.

For fluorescent stain, sections were incubated in secondary donkey anti-rabbit Alexa Fluor 488 for synaptophysin and Iba1 antibody and goat anti-chicken Alexa Fluor 488 for GFAP antibody (Invitrogen, Carlsbad, CA, USA) for 1 hour. Sections were then mounted and coverslipped with Fluoromount-G (Southern Biotech, Birmingham, AL, USA).

**Quantitative analyses**

The biochemical data were quantitatively analyzed using Image J 1.36b software. For synaptophysin quantification, fluorescent sections were imaged with a Leica DM 2500 laser scanning confocal with identical laser and detection settings. Gray-scale images (5 sections per animal, n=6) were analyzed using Image J software.

Microglia and astroglia were modeled using Bitplane Imaris software and changes in these cells, such as cell body area, process length, and number of microglial branches were analyzed (5 sections per animal, n=6).

**Proinflammatory ELISA**

One V-Plex proinflammatory panel 1 (mouse) kit for IFN-γ, IL-1β, IL-2, IL-4, IL-6, KC/GRO, IL-10, IL-12p70, TNF-α (MSD, Meso Scale Discovery) was used. Soluble fractions from hippocampus were diluted with diluent 41 (2-fold dilution). 50 μl per well of prepared sample, calibrator and control were added in a plate and were incubated at room temperature for 2 hours. Then, samples were washed and incubated with the detection antibody solution for 2 hours at room temperature. After washes, 150 μl of Read buffer were added to each well and the plate was read on the MSD instrument. The data obtained were normalized with the protein content of each sample.

For soluble fraction extraction, the hippocampus was homogenized (using a Dounce homogenizer) in T-PER^TM^ (Tissue Protein Extraction Reagent) (Thermo Scientific) containing complete protease and phosphatase inhibitor cocktails (Sigma). After homogenization, the soluble fraction was isolated by one centrifugation (100000xg, 1 h at 4°C). The protein content of soluble fraction was determined using the Bradford assay.

**Statistical analyses**

After confirming normal distribution, all data were analyzed by Student’s *t*-test comparisons and one-way or two-way analysis of variance (ANOVA), followed by Tukey’s comparisons using GraphPad Prism 5^®^ software (GraphPad Prism Inc., San Diego, CA, USA). The significance was set at 95% of confidence. All values are presented as mean ± SEM.

**Supplemental Figures**

***Figure S1. Diabetic characterization for type 1 diabetes animal groups.*** A) STZ treated mice (Ntg/STZ and htau/STZ groups) displayed a reduction in body weight since the injection week until the sacrifice (Two-way ANOVA: trials [F(6,462)=11.87, p<0.0001], treatment [F(3,462)=157.3, p<0.0001], and interaction [F(18,462)=3.076, p<0.0001], Tukey’s multiple comparisons test, ***p<0.001. * significance versus Ntg, and # significance versus htau; blue for Ntg/STZ, and red for htau/STZ. n=14-21 per group). B) Glucose level measured 3 days after STZ injection (1^st^ measure) and before sacrifice (2^nd^ measure) showed a significant increase in glucose in STZ-treated mice compared to Ntg and htau. (Two-way ANOVA: trials [F(1,86)=66.17, p<0.0001], treatment [F(3,86)=49.38, p<0.0001], and interaction [F(3,86)=0.4446, p=0.7217], Tukey’s multiple comparisons test, ****p<0.001. n=8-15 per group). C) A1c levels measured before sacrificed revealed a significant increase in STZ-treated groups (One-way ANOVA, ****p<0.0001, F(3,17)=23.71, Tukey’s multiple comparisons test, ***p<0.001. n=5-6 per group). D) Insulin concentration in blood samples measured by ELISA displayed a reduction in STZ-treated groups (One-way ANOVA, ***p<0.0003, F(3,25)=9.263, Tukey’s multiple comparisons test, ***p<0.001, **p<0.01. n=4-12 per group). E) Immunoblot analyses of insulin receptor (IR) and phosphor-insulin receptor (pIR) of protein extracts from hippocampal membrane fraction of Ntg, Ntg/STZ, htau, and htau/STZ mice are shown in alternating lanes (E1). E2) Quantification normalized to β-tubulin, and expresses as relative units, for pIR and IR. Streptozotocin does not alter IR levels but reduce the receptor phosphorylation (One-way ANOVA, *p=0.0279, F(3,17)=3.877, Tukey’s multiple comparisons test, *p<0.05. n=5 per group). The values represent means ± SEM.

***Figure S2. Diabetic characterization for type 2 diabetes animal groups.*** db/db and db/db-htau mice displayed a high weight (A) (n=9-19 per group), glucose (B) (n=8-18 per group), and A1c levels before the treatment (C) (n=8-11 per group) at 10 months of age. However, the insulin concentration measured in plasma (D) was low in both groups (n=4 per group). No significant differences were detected between both groups for these measures. E) Immunoblot analyses of insulin receptor (IR) and phosphor-insulin receptor (pIR) of protein extracts from hippocampal membrane fraction of Ntg, db/db, and db/db-htau mice are shown in alternating lanes (E1). E2). Quantification normalized to β-tubulin, and expresses as percentage of control, for pIR and IR. Diabetic condition does not alter IR, but reduce pIR levels for type 2 diabetes mice (One-way ANOVA, **p=0.0036, F(3,18)=6.498, Tukey’s multiple comparisons test, *p<0.05. n=4-8 per group). F-G) Diabetic groups (db/db and db/db-tauKO mice) showed a significant increase in A1c levels (F) and insulin concentration (G) before sacrifice at 8 weeks (F: One-way ANOVA, ****p<0.0001, F(3,32)=29.24, Tukey’s multiple comparisons test ***p<0.001, **p<0.01. n=7-12 per group. G: One-way ANOVA, **p=0.0025, F(3,13)=8.276, Tukey’s multiple comparisons test *p<0.05. n=3-7 per group). The values represent means ± SEM.

***Figure S3. Synaptophysin and profilin changes in T1DM mice.*** Immunoblot analyses of synaptophysin (Syn) and profilin in hippocampal synaptosomes of Ntg, Ntg/STZ, htau and htau/STZ mice are shown in alternating lanes (A1). A2) Quantification normalized to β-tubulin, and expresses as relative units, showing a reduction in levels of synaptohpysin and profilin but without significant differences (n=6 per group). The values represent means ± SEM.

***Figure S4. Inflammatory profile in T1DM mice.*** A) MSD cytokines analysis in Ntg, Ntg/STZ, htau and htau/STZ mice revealed no differences among the groups of mice in the levels of the cytokines analyzed (n=10 per group). The values represent means ± SEM.

***Figure S5. Genetic characterization of type 2 diabetes mice models.*** A) We used a genetic-gain-of function by crossing the db/db mice with the htau mice, generating the db/db-htau mice (A1). Immunohistochemistry for HT7 revealed that htau (A2) and db/db-htau (A3) mice accumulates tau in the pyramidal cells of the hippocampus. B) In order to ablate tau in the type 2 diabetes mice, we crossed the db/db mice to tauKO mice and we generated homozygous db/db-tauKO mice (B1). We assessed the tau expression by WB (B2), demonstrating a significant reduction in tau levels (B3) in the tauKO and db/db-tauKO mice (One-way ANOVA, ****p<0.0001, F(3,20)=23.52, Tukey’s multiple comparisons test, ***p<0.001, n=6 per group). The values represent means±SEM. Scale bars: 250 μm. so: stratum oriens, sp: stratum pyramidale, sr: stratum radiatum, slm: stratum lacunosum-moleculare.

***Figure S6. Increased tau phosphorylation in db/db-htau mice.*** AT8 immunostaining in db/db (A) and db/db-htau (B) mice revealed an increase in tau phosphorylation (AT8) in db/db-htau mice, where we observed tau positive neurons (black arrows) which were absent in htau mice. Scale bars: 250 μm. so: stratum oriens, sp: stratum pyramidale, sr: stratum radiatum, slm: stratum lacunosum-moleculare.

***Figure S7. Timeline for T1DM and T2DM conditions.*** A) Type 1 diabetes animal groups (Ntg, Ntg/STZ, htau, and htau/STZ). Ntg/STZ and htau/STZ animals received 2 intraperitoneal STZ injections (75mg/Kg) at 14 months. After three days, the glucose level was measured to corroborate that diabetes condition was induced. Behavioral test was performed at 15 months-old and two weeks later, animals were sacrificed. B) For type 2 diabetes genetic gain-of-function approach (Ntg, htau, db/db, and db/db-htau), behavioral tests were performed at 10 months, after which the animals were sacrificed. C) For type 2 diabetes with tau ablation (Ntg, tauKO, db/db, and db/db-tauKO), MWM was performed at 8 weeks, after which animals were sacrificed.

***Figure S8. Synaptosome isolation protocol.*** A) The hippocampus was homogenized in DEPC-treated water supplemented with 0.32 M sucrose, 20 mM Tris-HCl, 0.5 M EDTA and 0.5 M EGTA (pH 7.4), containing complete protease and phosphatase inhibitor cocktails. After homogenization, we made two sequential centrifugations at 4°C: 7000 rpm for 10 minutes and 17000 rpm for 20 min. B) Western blot analysis demonstrated that the syanptosome (SN) contain synaptic markers (Syn, PSD-95) with low or nothing of nuclear (NeuN) or glial (Iba1 and GFAP) markers which were more abundant in the soluble cytosolic fraction (S).
